# Comparison of Clinical Outcomes Using “Elevate Anterior” versus “Perigee” System Devices for the Treatment of Pelvic Organ Prolapse

**DOI:** 10.1155/2015/479610

**Published:** 2015-03-29

**Authors:** Cheng-Yu Long, Chiu-Lin Wang, Ming-Ping Wu, Chin-Hu Wu, Kun-Ling Lin, Cheng-Min Liu, Eing-Mei Tsai, Ching-Ju Shen

**Affiliations:** ^1^Department of Obstetrics and Gynecology, Kaohsiung Medical University Hospital, Kaohsiung Medical University, 100 Ziyou 1st Road, Kaohsiung City 80756, Taiwan; ^2^Department of Obstetrics and Gynecology, Kaohsiung Municipal Hsiao-Kang Hospital, Kaohsiung Medical University, 100 Ziyou 1st Road, Kaohsiung City 80756, Taiwan; ^3^Department of Obstetrics and Gynecology, Chi Mei Foundation Hospital, Tainan, Taiwan

## Abstract

*Objective.* This study aims to compare clinical outcomes using the Perigee versus Elevate anterior devices for the treatment of pelvic organ prolapse (POP). *Study Design.* One hundred and forty-one women with POP stages II to IV were scheduled for either Perigee (*n* = 91) or Elevate anterior device (*n* = 50). Preoperative and postoperative assessments included pelvic examination, urodynamic study, and a personal interview about quality of life and urinary symptoms. *Results.* Despite postoperative point C of Elevate group being significantly deeper than the Perigee group (median: −7.5 versus −6; *P* < 0.01), the 1-year success rates for two groups were comparable (*P* > 0.05). Apart from urgency incontinence, women with advanced POP experienced significant resolution of irritating and obstructive symptoms after both procedures (*P* < 0.05), generating the improvement in postoperative scores of Urogenital Distress Inventory (UDI-6) and Incontinence Impact Questionnaire (IIQ-7) (*P* < 0.01). On urodynamics, only the residual urine decreased significantly following these two procedures (*P* < 0.05). Women undergoing Perigee mesh experienced significantly higher visual analogue scale (VAS) scores and vaginal extrusion rates compared with the Elevate anterior procedure (*P* < 0.05). *Conclusions.* With comparable success rates, the Elevate procedure has advantages over the Perigee surgery with lower extrusion rate and postoperative day 1 VAS scores.

## 1. Introduction

It has been estimated that a lifetime risk of undergoing primary surgery for pelvic organ prolapse (POP) or urine incontinence for a woman is about 11% [1]. Despite the fact that anterior colporrhaphies have been the empirical treatment of POP for a long time, it carries a higher failure rate [1]. Thus, surgery with mesh or graft materials has gained more and more popularity over the last decade due to the excellent short-term cure rate, especially in the anterior compartment [2, 3].

The Perigee (AMS, Inc., Minnetonka, MN, USA) system is one kind of the synthetic mesh kits recently launched and adopted in pelvic reconstruction for the treatment of anterior vaginal wall prolapse. The Elevate anterior (AMS, Inc., Minnetonka, MN, USA) device is the next generation of the Perigee system, claiming the use of a type I polypropylene Intepro Lite mesh and single vaginal incision in this newer procedure. The Perigee mesh is anchored using trocar passes through the obturator foramen and the latter mesh with hooks is reached with direct penetration to the obturator fascia and sacrospinous ligament [4, 5]. Therefore, the Elevate anterior mesh provides both anterior and apical support, which is important due to the notable reports of compensatory recurrence rates after POP surgery [6].

However, the United States Food and Drug Administration (FDA) announced a public health notification regarding “serious complications associated with transvaginal placement of surgical mesh in repair of POP and stress urinary incontinence (SUI)” in 2011 July [7]. Indeed, the FDA warning has caused further caution when selecting the type of mesh kits. We had published a pilot study comparing clinical outcomes using “Perigee and/or Apogee” versus “Prolift anterior and/or posterior” devices for the treatment of POP [8]. This time, we wonder whether the newer Elevate device offers a safer and less painful procedure and whether this would be more durable. In reviewing the literature, limited data are available on the comparisons of efficacy and safety between these two graft-reinforced POP surgeries. Accordingly, we evaluated our experience with the focus on safety, clinical outcomes, and urodynamic findings following these two procedures.

## 2. Materials and Methods

From June 2004 to December 2012, two hundred consecutive symptomatic women with stage II or greater anterior/apical compartment prolapse defined by the POP quantification (POP-Q) staging system [9] were referred for cystocele repair using Perigee or Elevate anterior system devices (120 Perigee; 80 Elevate devices) to our hospital. Concomitant anti-incontinence sling surgeries, including tension-free vaginal tape (TVT; Gynecare TVT, Ethicon, Inc., Piscataway, NJ, USA), TVT-O (Gynecare TVT-Obturator System, Ethicon, Inc., Somerville, NJ, USA), Monarc (AMS, Inc., Minnetonka, MN, USA), and Ajust (C.R. Bard, Inc., Murray Hill, NJ, USA), were performed in patients with current or occult urodynamic stress incontinence (USI). Fifty-nine women were excluded from our study due to various reasons, including incomplete medical records (*n* = 24; fifteen women in the Perigee group, nine in the Elevate group) and inadequacy or loss of follow-up (*n* = 35; twenty-five women in the Perigee group, ten in the Elevate group). Finally, the remaining 141 women were divided into the Perigee group (*n* = 91) and the Elevate group (*n* = 50).

Preoperative and postoperative assessments included pelvic examination using the POP-Q staging system, urodynamic study, and a personal interview to evaluate the short forms of Urogenital Distress Inventory (UDI-6), Incontinence Impact Questionnaire (IIQ-7) [10], and urinary symptoms with the standardized questionnaire taking into account the 2002 ICS definitions [11]. Women were asked to fill out the VAS (visual analogue scale) scores during the postoperative day 1 rounds. Urodynamic studies were performed according to the recommendations by the International Continence Society [12] with a 6-channel urodynamic monitor (MMS; UD2000, Enschede, Netherlands). USI was defined as involuntary urinary loss with cough in the absence of detrusor contractions during cystometry. The diagnosis of occult USI was made by the occurrence of urinary leakage during the reduction of prolapse. Any uninhibited detrusor contraction during filling cystometry was deemed positive for idiopathic detrusor overactivity (DO).

### 2.1. Operative Technique: Perigee Device

Firstly, an anterior vaginal vertical incision was opened from the bladder neck toward the anterior fornix of the vagina. After hydrodissection, the paravesical space was created on both sides in order to put the index finger behind the obturator foramen. During the Perigee procedure, superior trocars were inserted through the upper medial angle of the obturator foramen at the level of the clitoris, and the inferior trocars were inserted 2 cm inferior and 1 cm lateral to the upper incisions. All trocars were passed through the arcus tendineus fascia pelvis and emerge in the vaginal wound. The mesh arms of Perigee devices were attached to the corresponding passers and brought out of the skin wounds [4].

### 2.2. Operative Technique: Elevate Device

After hydrodissection and separation of the paravesical fascia and vaginal mucosa, the Elevate anterior mesh with bilateral upper and lower arms was pushed to the obturator fascia and sacrospinous ligaments, respectively. Graft anchorage was performed via self-fixating tips, which avoided blind trocar passage through the obturator and perirectal fossa seen in Perigee mesh kit techniques. Then, cystoscopy was performed to exclude any bladder injury and to confirm intact ureters.

The synthetic mesh was put under the bladder base and anchored with 2-0 PDS (polydioxanone) sutures proximally and distally. The vaginal wound was closed with 3-0 polyglactin sutures. The skin wounds were closed using Dermabond and vaginal packing was placed for 24–48 hours. Operative time was calculated from the first incision of anterior vaginal wall to the end of wound closure. All patients were administered antibiotics (intravenous Cefazolin 1 g; Cefamezin, Fujisawa, Tokyo, Japan) before operation. The surgeries were carried out with the women under spinal, epidural, or general anesthesia. All procedures were finished mainly by the first author (Cheng-Yu Long), with individual experience of over 400 transvaginal mesh repairs.

Postoperative follow-up was scheduled at 1, 3, 6, and 12 months and then yearly thereafter. All POP-Q measurements were made by the first author. Surgical failure was defined as the most distal portion of prolapse over stage II or more, regardless of primary or de novo site. The International Urogynecological Association (IUGA)/International Continence Society (ICS) scale is used for the classification of the mesh-related complications [13]. Ethical approval by the Institutional Review Board of our hospitals had been obtained for data analysis. Statistical calculations were performed using Student's *t*-test, Mann-Whitney *U* test, McNemar's or Wilcoxon signed rank test for continuous variables, and the chi-square or Fisher's exact test for categorical variables. A *P* < 0.05 was considered statistically significant.

We assessed the power of tests for differentiating the surgical outcome between groups, and power analysis showed that around 50–60 women in each group would have a power of 80%. Although some comparisons, such as DO rates, could not reach sufficient power due to the limited numbers of the Elevate group, we utilized multiple parameters of POP-Q system to evaluate the postoperative change. We found with >40 women in each group that there was a power of over 85% for discrimination.

## 3. Results

**Figure 1:**
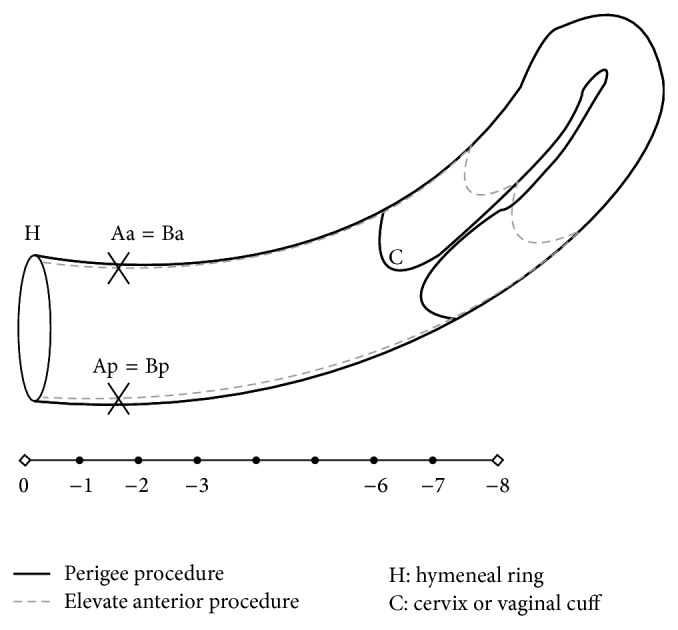
Postoperative point C of the Elevate group was significantly deeper than that in the Perigee group.

Participant characteristics of both groups are compared in Table 1. There was no difference between the two groups with regard to age, parity, current hormone use, diabetes, hypertension, prior history of hysterectomy or POP repair, POP stages, and concomitant procedures (*P* > 0.05). As for the POP-Q analysis, there was a significant improvement at points Aa, Ba, C, Ap, and Bp (*P* < 0.01) in both groups except for total vaginal length (*P* > 0.05; Table 2). Moreover, postoperative point C of the Elevate group was significantly deeper than that in the Perigee group (*P* < 0.01) (Figure 1). Other POP-Q points between the two groups did not differ significantly (*P* > 0.05). The 1-year success rate was comparable in both groups (*P* > 0.05; Table 2).

The prevalence of urinary symptoms in both groups, including urinary frequency, SUI, incomplete bladder emptying, urinary hesitancy, and nocturia, was found to be significantly lower following surgery (*P* < 0.01). As expected, postoperative UDI-6 and IIQ-7 scores of both groups improved in a significant manner (*P* < 0.01). However, the rate of urgency urinary incontinence did not show statistical significance in both groups (*P* > 0.05) (Table 3).

The percentage of DO decreased significantly postoperatively only in the Perigee group (*P* = 0.002; Table 4), but this was not the case in the Elevate group (*P* > 0.05; Table 4). As for urodynamic parameters, including maximum flow rate, residual urine, maximum cystometric capacity, functional urethral length, maximum urethral closure pressure, and urethral closure area, they did not show significant changes in either group (*P* > 0.05). Only the residual urine decreased significantly following these two procedures (*P* < 0.05; Table 4).

The Perigee procedure had a shorter operative time than did the Elevate procedure, but this did not reach significant difference (*P* = 0.07). With regard to intraoperative complications including bladder injury, rectal injury, and transfusion, none was suspected and confirmed by cystoscopic and rectal examinations. All postoperative complications in both groups included urinary tract infection (11.7% versus 16.7%), voiding dysfunction (difficulty initiating the void) (3.3% versus 2.1%), pelvic hematoma in none (0%), and dyspareunia (16.5% versus 12%). Chi-square and Fisher's exact tests showed no significant differences between both devices in intraoperative and postoperative comparisons (*P* > 0.05). However, women undergoing Perigee mesh experienced significantly higher VAS scores (3.9 ± 0.8 versus 2.6 ± 1.5; *P* < 0.01) (Table 5) compared with the Elevate anterior procedure.

The rate of vaginal extrusion was borderline significantly higher in the Perigee group (10/91; 11% versus 1/50; 2%; *P* = 0.05) (Table 5). All patients were initially treated conservatively with estrogen vaginal cream. However, eight of them (seven in the Perigee group, one in Elevate group) subsequently needed excisions of the exposed mesh. One woman with Perigee mesh had persistent extrusion requiring repeat excision after 3 months. According to the mesh-related complications by ICS/IUGA scale, we found that one woman of Elevate group was 3BT3S1, and the remaining 10 women of Perigee group were as follows: two with 2BT2S1, two with 2BT3S1, five with 2AT3S1, and one with 7AT2S3.

## 4. Discussion

The surgical efficacy of anatomical correction was comparable in both groups at 1-year follow-up. Although earlier launch of the Perigee device had the longer follow-up time in our series, we only reviewed its postoperative 1-year POP-Q data to match the Elevate group. The 1-year success rate of 94% for our Elevate anterior group is slightly higher than another study by Rapp et al. [5], but lower than the data of Lo et al. [14]. Both reported the 90.5% cure rates at 2-year follow-up and 96.9% at 1-year follow-up, respectively. This may be due to the different study periods and subjects. We found that younger (mean age of 61.7 years) and slimmer (mean BMI of 24) women were enrolled in our study.

With insignificant difference of total vaginal length, we found that postoperative point C of the Elevate group was significantly deeper than that in the Perigee group, in accordance with another study [14]. A possibility was that anchorage of lower arms of the Elevate anterior mesh to sacrospinous ligament provided better postoperative support of the apical compartment. However, it is hard to draw a conclusion due to the shorter follow-up time and limited case numbers.

With the advance of mesh materials for the treatment of anterior vaginal prolapse, synthetic mesh has the advantages of durability and strength over traditional anterior colporrhaphy [3]. However, a number of reported mesh complications between 2008 and 2010 provoked a series of actions by the FDA that resulted in reclassifying mesh kits to class III devices and requiring these manufacturers of these commercial kits to conduct research studies about their device if they desired to continue selling their devices in the market [7]. The Elevate anterior system device was launched in November 2010, and it is the next generation of the Perigee system. We questioned whether the newer Elevate device provides a safer, more durable, and less painful procedure; therefore, we included these two former and latter marketed kits of the same manufacturer for comparison in this study.

Lo et al. found that Elevate mesh caused a significantly higher rate of de novo SUI postoperatively than did the Perigee device [14], but we did not meet this condition. A possibility was that over half (60.3%; 85/141) of our women underwent concomitant anti-incontinence surgery. Interestingly, a recent study showed that the rate of overactive bladder (OAB) symptoms may be higher significantly when TVT and POP repair were performed together [15]. We did not find these results. A possibility was that most of our women underwent the minisling or transobturator tape rather than TVT procedure. The horizontal shape of the minisling or TOT tape may be less obstructive [16, 17] and unlikely to cause irritating symptoms. However, whether the TOT is more suitable in women with preoperative OAB symptoms remains unclear.

It is well known that severe POP could cause bladder outlet obstruction by urethral kinking or external compression [18], which can promote uninhibited detrusor contractions. Our findings of postoperative improvement in irritating and obstructive symptoms after mesh repair might be partly related to the release of urethral obstruction. This also contributed to the improvement of DO and urogenital distress questionnaires in a significant manner. Despite the changes in DO of Elevate patients not being significant, larger sample size may shed more light upon this condition.

In theory, lower uroflow rate and higher postvoid residual urine on urodynamics could be improved after the POP surgery. However, we did not find any significant change in all urodynamic parameters postoperatively except for the decrease of residual urine, in accordance with our previous study [19]. Urodynamic study might not have been sensitive enough to identify minimal changes in all dysfunctional voiding. Another possibility was that not every woman experienced stage IV advanced POP in this study.

The use of blind trocars of commercial kits has arisen some concerns [7]. A review study revealed a total of 1.9% of visceral injury during anterior and/or posterior 2nd-generation mesh repair [20]. Thus, the newer Elevate mesh is designed for no blind or external trocar passage potentially minimizing the risk of tissue trauma due to its single incision procedure. We were fortunate to avoid these complications completely in both groups. Besides, the Perigee procedure had shorter but not significant operative time than the Elevate procedure, indicating that the authors may be more comfortable with the former mesh as a result of longer experience.

Mesh extrusion refers to the mesh pushing itself out of the tissue and producing a protrusion, whereas mesh erosion refers to the destruction of the vaginal layer covering the mesh, which usually migrates into the vaginal lumen [21]. In theory, the less the amount of mesh placed, the lower the rate of extrusion occurring. As expected, the vaginal extrusion rate for the Perigee group (11%; 10/91) was higher than the Elevate group (2%; 1/50), this possibly being related to the use of a type I Intepro Lite mesh with lower molecular weight in this newer procedure. Lo et al. also obtained similar results (4.9% for Perigee and 0% for Elevate group) [14]. In addition, we found another advantage of the Elevate device is that all needles pass through a single vaginal incision, noticeably minimizing its postoperative pain scores.

We found the conservative management of vaginal extrusion was disappointing in this study. Although all women received initial treatment with estrogen vaginal cream, over half (55.6%; 6/11) subsequently needed excision of the exposed mesh. A previous study concluded that mesh extrusion after prolapse surgery is more likely to occur with concomitant hysterectomy [22]. Similar results were obtained in our study; four mesh extrusions occurred in Perigee patients undergoing concomitant hysterectomy (4/17; 23.5%) and another 6 in Perigee women without hysterectomy (6/74; 8.1%).

A flaw of this study was that heterogeneous study population included women having more than one compartment repair and those with and without anti-incontinence sling surgery. However, it would be difficult to select a patient population undergoing anterior mesh alone. In conclusion, the results of our study suggested that Perigee and Elevate anterior devices for POP repair had comparable success rates and intraoperative complications. However, the Elevate procedure has advantages over the Perigee surgery with lower extrusion rate and postoperative day 1 VAS scores. Although the Elevate anterior mesh created a deeper anatomical position of cervix or vaginal cuff, it did not appear to have a significant impact on functional outcome. Women with advanced prolapse could experience significant improvement of obstructive and irritating symptoms after transvaginal mesh repair. More prospective randomized studies and longer follow-up time of functional and anatomical results are urgently needed to guide the appropriate use of mesh by gynecologists and urologists in the future.

## Figures and Tables

**Table 1:** Clinical background of patients with pelvic organ prolapse in both groups. Data are given as mean ± standard deviation, median [range], or *n* (%).

	Perigee (*n* = 91)	Elevate (*n* = 50)	*P* values
Mean age (years)	61.0 ± 12.0	62.9 ± 9.8	0.32^∗^
Mean parity	3.4 ± 1.3	3.3 ± 1.3	0.72^∗^
Mean BMI (kg/m^2^)	23.7 ± 3.5	24.5 ± 3.7	0.21^∗^
Menopause	72 (79.1)	44 (88.0)	0.19^∗∗^
Current hormone therapy	5 (5.5)	4 (8.0)	0.72^∧^
Diabetes mellitus	10 (11.0)	9 (18.0)	0.14^∗∗^
Hypertension	20 (22.0)	18 (36.0)	0.07^∗∗^
History of hysterectomy	9 (9.9)	10 (20.0)	0.09^∗∗^
History of POP repair	3 (3.3)	5 (10.0)	0.13^∧^
POP			
Stage 2	20 (22.0)	10 (20.0)	0.78^∗∗^
Stage 3	66 (72.5)	38 (76.0)	0.65^∗∗^
Stage 4	5 (5.5)	2 (4.0)	1.0^∧^
Concomitant procedures			
Posterior repair	3 (3.3)	2 (4.0)	1.0^∧^
Vaginal hysterectomy	17 (18.7)	16 (32.0)	0.07^∗∗^
Midurethral sling	58 (63.7)	27 (54.0)	0.26^∗∗^

BMI, body mass index; POP, pelvic organ prolapse; ^∗^Student's *t*-test; ^∗∗^Chi-square test; ^∧^Fisher's exact test.

**Table 2:** Pelvic organ prolapse quantification (POP-Q) values in both groups before and 1 year after surgery. Data are given as median (range) or *n* [%].

POP-Q parameters (cm)	Perigee (*n* = 91)			Elevate (*n* = 50)			*P* values^#^
	Pre-op	Post-op	*P* values^∗^	Pre-op	Post-op	*P* values^∗^	
Aa	2 (0~3)	−2 (−1~−3)^#^	**<0.001**	1.0 (−2~3)	−2 (−3~−1.5)^#^	**<0.001**	**0.50^#^**
Ba	2.5 (−1~4)	−2 (−1~−5)^#^	**<0.001**	2.0 (−1~4)	−2 (−3~−1.5)^#^	**<0.001**	**0.19^#^**
C	−1 (−3~5)	−6 (−5~−8)^#^	**<0.001**	−2 (−5~2)	−7.5 (−9~−6)^#^	**<0.001**	**<0.01^#^**
Ap	−1 (−2~3)	−2 (−3~0)^#^	**<0.001**	−2 (−3~0)	−2 (−3~−2)^#^	**0.006**	**0.22^#^**
Bp	−1 (−2~4)	−2 (−5~0)^#^	**<0.001**	−2 (−2~1)	−2 (−3~−2)^#^	**<0.001**	**0.38^#^**
Tvl	8 (5~9)	8 (6~9)^#^	**0.54**	8 (7~10)	8.5 (8~10.5)^#^	**0.90**	**0.09^#^**
Success rate		85 [93.4]			47 [94]		**1.0^**∗∗**^**

Pre-op, preoperative; Post-op, postoperative; Tvl, total vaginal length.

^∗^Wilcoxon signed rank test; ^#^Mann-Whitney *U* test; ^∗∗^Fisher's exact test.

**Table 3:** Urinary symptoms of patients with pelvic organ prolapse in both groups before and 6 months after surgery. Data are given as *n* (%).

Symptoms	Perigee (*n* = 91)			Elevate (*n* = 50)		
	Pre-op	Post-op	*P* value	Pre-op	Post-op	*P* value
Urinary frequency	51 (56.0)	16 (17.6)	<0.01^∗^	24 (48)	5 (10)	<0.01^∗^
SUI	59 (64.8)	16 (17.6)	<0.01^∗^	32 (64)	7 (50)	<0.01^∗^
UUI	35 (38.5)	27 (29.7)	0.06^∗^	14 (28)	11 (22)	0.38^∗^
Incomplete emptying	65 (71.4)	11 (12.1)	<0.01^∗^	37 (74)	9 (18)	<0.01^∗^
Urinary hesitancy	53 (58.2)	9 (9.9)	<0.01^∗^	31 (62)	7 (14)	<0.01^∗^
Nocturia	53 (58.2)	43 (47.3)	0.041^∗^	39 (78)	28 (56)	<0.01^∗^
UDI-6	23.0 ± 9.9	5.7 ± 1.8	<0.01^∗∗^	19.7 ± 9.5	7.9 ± 3.4	<0.01^∗∗^
IIQ-7	27.0 ± 13.6	4.8 ± 1.9	<0.01^∗∗^	28.3 ± 15.3	6.3 ± 3.1	<0.01^∗∗^

SUI, stress urinary incontinence; UUI, urgency urinary incontinence; UDI-6, Urogenital Distress Inventory; IIQ-7, Incontinence Impact Questionnaire.

^∗^McNemar's test; ^∗∗^Paired *t*-test.

**Table 4:** Urodynamic changes in both groups before and 6 months after surgery. Data are given as *n* (%) or mean ± standard deviation.

Parameters	Perigee (*n* = 91)			Elevate (*n* = 50)		
	Pre-op	Post-op	*P* value	Pre-op	Post-op	*P* value
DO	31 (34.1)	12 (13.2)	0.002^∗∧^	17 (34.0)	11 (22.0)	0.29^∗^
Qmax (mL/s)	18.6 ± 9.0	19.6 ± 10.2	0.26^∗∗^	21.0 ± 14.3	18.0 ± 8.9	0.06^∗∗^
RU (mL)	63.1 ± 45.3	32.8 ± 32.7	0.015^∗∗∧^	71.3 ± 50.3	36.6 ± 22.6	0.038^∗∗∧^
FS (mL)	164.6 ± 73.9	154.5 ± 64.3	0.23^∗∗^	135.7 ± 54.9	133.0 ± 59.5	0.79^∗∗^
MCC (mL)	409.5 ± 127.0	400.7 ± 111.4	0.35^∗∗^	332.5 ± 81.1	324.8 ± 109.4	0.57^∗∗^
Pdet (cmH_2_O)	26.5 ± 19.9	28.0 ± 17.9	0.52^∗∗^	19.5 ± 7.6	18.6 ± 11.0	0.68^∗∗^
FUL (mm)	24.6 ± 6.1	27.1 ± 4.9	0.07^∗∗^	27.8 ± 7.1	25.4 ± 8.4	0.14^∗∗^
MUCP (cmH_2_O)	56.3 ± 26.2	63.1 ± 34.2	0.25^∗∗^	73.0 ± 29.5	72.5 ± 24.9	0.90^∗∗^
UCA (mmcmH_2_O)	730.3 ± 456.9	816.5 ± 408.3	0.12^∗∗^	1012.6 ± 396.8	934.4 ± 391.8	0.11^∗∗^

DO, detrusor overactivity; Qmax, maximum flow rate; RU, residual urine; FS, first sensation to void; MCC, maximum cystometric capacity; Pdet, detrusor pressure at peak flow; FUL, functional urethral length; MUCP, maximum urethral closure pressure; UCA, urethral closure area.

^∗^McNemar's test; ^∗∗^Paired *t*-test.

^∧^Statistical significance.

**Table 5:** Intraoperative, postoperative, and mesh-related complications of patients with pelvic organ prolapse in both groups. Data are given as *n* (%).

	Perigee (*n* = 91)	Elevate (*n* = 50)	*P* values
Intraoperative complications			
Operative time (minutes)	35.3 ± 16.1	45.2 ± 25.3	0.07^#^
Bladder injury	0	0	
Rectal injury	0	0	
Blood transfusion	0	0	
Postoperative complications			
Post-op day 1 VAS scores	3.9 ± 0.8	2.6 ± 1.5	<0.01^#^
Urinary tract infection	9 (11.7)	8 (16.7)	0.29^∗^
Voiding dysfunction	2 (3.3)	4 (2.1)	0.19^∗∗^
Perineal hematoma	0	0	
Dyspareunia	15 (16.5)	6 (12)	0.25^∗^
Mesh complications			
Vaginal extrusion	10 (11)	1 (2)	0.05^∗∗^
Bladder extrusion	0	0	

Post-op, postoperative; VAS, visual analogue scale.

^#^Student's *t*-test; ^∗^Chi-square test; ^∗∗^Fisher's exact test.
